# A generalized radial integration by parts formula and its applications to Caffarelli–Kohn–Nirenberg inequalities

**DOI:** 10.1007/s13324-025-01060-y

**Published:** 2025-04-26

**Authors:** Giovanni Di Fratta, Alberto Fiorenza

**Affiliations:** 1 Dipartimento di Matematica e Applicazioni “R. Caccioppoli”, Università degli Studi di Napoli “Federico II”, Via Cintia, Complesso Monte S. Angelo, 80126 Naples, Italy; 2 Dipartimento di Architettura, Università degli Studi di Napoli “Federico II”, Via Monteoliveto, 80134 Napoli, Italy; 3 Istituto per le Applicazioni del Calcolo “Mauro Picone”, sezione di Napoli, Consiglio Nazionale delle Ricerche, via Pietro Castellino, 111, 80131 Napoli, Italy

**Keywords:** Caffarelli–Kohn–Nirenberg (CKN) inequalities, Radial integration by parts formula, Sobolev inequality, Hardy inequality, 49J40, 46E35, 35B06, 26D10

## Abstract

This paper builds upon the Caffarelli–Kohn–Nirenberg (CKN) weighted interpolation inequalities, which are fundamental tools in partial differential equations and geometric analysis for establishing relationships between functions and their gradients when power weights are involved. Our work broadens the scope of these inequalities by generalizing them to encompass a broader class of radial weights and exponents. Additionally, we extend the application of these inequalities to the class $C^{\infty } ( {\overline{\Omega }})$ of smooth functions defined on bounded domains with Lipschitz boundaries. To achieve this generalization, we formulate a new integration by parts formula that accounts for more general weights, a wider range of exponents, and $C^{\infty }({\overline{\Omega }})$ functions. The resulting generalized CKN-type inequalities offer explicit upper bounds on the optimal constants, independent of the domain’s geometry, consistent with the scaling invariant nature of the inequalities.

## Introduction

The Caffarelli–Kohn–Nirenberg (CKN) weighted interpolation inequalities, introduced in their paper from 1984  [7], have significantly impacted mathematical analysis. By incorporating power weights, these inequalities establish fundamental relationships between the integrability properties of functions and their gradients, thus extending classical results and providing powerful tools for a priori estimates of solutions to partial differential equations (PDEs). The primary aim of [7] is a systematic analysis of the conditions under which the following family of first-order interpolation inequalities with weights is valid for any function $u \in C_c^{\infty }( {\mathbb {R}}^{N}),$ $N \geqslant 1$,

1.1 $$\begin{aligned} \left( \int _{{\mathbb {R}}^{N}} | x |^{\gamma r} |u|^{r} \textrm{d}x \right) ^{1 / r} \leqslant C \left( \int _{{\mathbb {R}}^{N}} | x |^{\alpha p} \left| \nabla u \right| ^{p} \textrm{d}x \right) ^{a / p} \left( \int _{{\mathbb {R}}^{N}} | x |^{\beta q} |u|^{q} \textrm{d}x \right) ^{(1 - a) / q}, \end{aligned}$$

where the real parameters $a, p, q, r, \alpha , \beta , \gamma$, are assumed to be subject to the following constraints

1.2 $$\begin{aligned}  &   p, q\geqslant 1, \quad r> 0, \quad 0 \leqslant a \leqslant 1, \quad \alpha , \beta , \gamma \in {\mathbb {R}}, \end{aligned}$$

1.3 $$\begin{aligned}  &   \gamma r+ N> 0, \quad \alpha p+ N> 0, \quad \beta q+ N> 0, \end{aligned}$$

and $C > 0$ is a constant that depends only on the involved parameters.

### Remark

When writing our estimates or inequalities, we will use either the extended integral notation as shown in (1.1) or the more concise norm notation associated with Lebesgue spaces. Our choice will be based on what we think will be more suitable and readable in that particular context. For instance, in norm notation, inequalities (1.1) assume the more familiar form found in [7]:

$$\begin{aligned} \left\| \, | x |^{\gamma } | u | \right\| _{L^{r}( {\mathbb {R}}^{N})} \leqslant C \left\| \, | x |^{\alpha } \left| \nabla u \right| \right\| _{L^{p}( {\mathbb {R}}^{N})}^a \cdot \Vert \; | x |^{\beta } | u | \;\Vert _{L^{q} ( {\mathbb {R}}^{N})}^{1 - a}. \end{aligned}$$

The validity of these inequalities, as shown in [7] through a scaling argument, hinges on the *dimensional balance equation*:

1.4 $$\begin{aligned} \frac{1}{r} + \frac{\gamma }{N} = a \left( \frac{1}{p} + \frac{\alpha - 1}{N} \right) + (1 - a) \left( \frac{1}{q} + \frac{\beta }{N} \right) . \end{aligned}$$

Precisely, it is shown in [7] that under the constraints (1.3) the first-order interpolation inequalities (1.1) are verified for every $u \in C_c^{\infty } ( {\mathbb {R}}^{N} )$ if, and only if, the balance equation (1.4) is satisfied and the exponent $\gamma$ is of the form

1.5 $$\begin{aligned} \gamma = a \sigma + (1 - a) \beta , \end{aligned}$$

for some real parameter $\sigma \in {\mathbb {R}}$ (not necessarily positive), which is bounded above by $\alpha$:

1.6 $$\begin{aligned} \sigma \leqslant \alpha , \end{aligned}$$

and also bounded below by $\alpha - 1$ when, formally, one sets $a = 1$ in the dimensional balance equation (1.4), i.e.,

1.7 $$\begin{aligned} \alpha - 1 \leqslant \sigma \leqslant \alpha \quad \text {when} \quad \frac{1}{r} + \frac{\gamma }{N} =\frac{1}{p} + \frac{\alpha - 1}{N} . \end{aligned}$$

Furthermore, it is established that for any compact subset of the parameter space where the conditions (1.2), (1.3), and (1.4) are satisfied and $\alpha - 1 \leqslant \sigma \leqslant \alpha$, the positive constant *C* in (1.1) is bounded, though [7] does not provide an explicit expression for a possible constant *C* in (1.1) that would make evident its qualitative behavior in terms of the involved parameters.

These inequalities generalize several classical results, including the Sobolev and Hardy inequalities, demonstrating their wide applicability and unifying role within the domains of PDEs and geometric analysis. Specifically, when $a = 1$ relations (1.4) and (1.5) give that (1.7) is satisfied and $\sigma = \gamma$. Therefore, when $a = 1$, the CKN inequalities (1.1) reduces to the Hardy-Sobolev inequalities [8, 10, 11, 23]

1.8 $$\begin{aligned} \left( \int _{{\mathbb {R}}^{N}} | x |^{\gamma r} |u|^{r} \textrm{d}x \right) ^{1 / r} \leqslant C \left( \int _{{\mathbb {R}}^{N}} | x |^{\alpha p} \left| \nabla u \right| ^{p} \textrm{d}x \right) ^{1 / p}, \end{aligned}$$

with the parameters satisfying the relations $p\geqslant 1$, $\gamma > - N/ r$, $\alpha > - N/ p$, $\alpha \geqslant \gamma \geqslant \alpha - 1$ and $r> 0$ given by

1.9 $$\begin{aligned} r= \frac{pN}{p(\alpha - \gamma ) + N- p}, \end{aligned}$$

The Hardy-Sobolev inequalities (1.8) thus interpolate between the usual Sobolev inequality ($\alpha = \gamma = 0$)

1.10 $$\begin{aligned} \left( \int _{{\mathbb {R}}^{N}} |u|^{r} \textrm{d}x \right) ^{1 / r} \leqslant C \left( \int _{{\mathbb {R}}^{N}} \left| \nabla u \right| ^{p} \textrm{d}x \right) ^{1 / p}, \end{aligned}$$

with $r= p^{*} :=\frac{pN}{N- p}$ being the Sobolev conjugate of $p$, and the weighted Hardy inequality when $\alpha = \gamma + 1$

1.11 $$\begin{aligned} \left( \int _{{\mathbb {R}}^{N}} | x |^{\gamma p} |u|^{p} \textrm{d}x \right) ^{1 / p} \leqslant C \left( \int _{{\mathbb {R}}^{N}} | x |^{(\gamma + 1) p} \left| \nabla u \right| ^{p} \textrm{d}x \right) ^{1 / p} . \end{aligned}$$

Further extensions of (1.11) are considered in [13], where it is also shown that one can take $C :=p/ \left( N+ \gamma p\right)$. We also note that Gagliardo-Nirenberg and Nash inequalities are particular cases of (1.1); we refer the reader to [4] for further insights into various parameter regimes and to [22] for a detailed exposition of Nirenberg’s original proof of the Gagliardo-Nirenberg inequality.

The Caffarelli–Kohn–Nirenberg (CKN) inequalities, along with their particular instances, such as the Hardy-Sobolev inequalities (1.8), provide a comprehensive framework for understanding the relationship between weights and geometric parameters in functional inequalities. More detailed insights into their broad scope can be gained through in-depth studies aimed at determining optimal constants, investigating the existence and properties of extremal functions within specific parameter regimes (see [12, 27]), and looking for further generalizations that encompass a broader range of kernels.

The primary objective of this paper is to present CKN inequalities that include more general kernels and extend to the class $C^{\infty } ( {\overline{\Omega }})$ of smooth functions defined on bounded domains with Lipschitz boundaries. To achieve this, we introduce a novel integration by parts formula that incorporates more general weights and permits potential regularity loss at the boundary. This extension yields generalized CKN-type inequalities that provide explicit upper bounds for optimal constants, which are independent of the domain’s geometry and preserve scaling invariance.

### State of the art

The Caffarelli–Kohn–Nirenberg (CKN) inequalities were first introduced in the seminal work of Caffarelli, Kohn, and Nirenberg in 1982 [6], specifically for the parameter choice $p= q= 2$ and $N= 3$. Their paper [6] deals with the partial regularity of so-called *suitable* weak solutions of the Navier–Stokes equations, whose existence was first proved by Scheffer in [32] when no externally applied forces were present and then extended to include this contribution in the Appendix of [6]. The partial regularity theorem provided therein concerns a parabolic analog of the Hausdorff dimension of the singular set. It remains a cornerstone in the quest to determine whether the Navier–Stokes equations yield a deterministic description of the flow of viscous incompressible fluids [20, 25, 36]. The weighted interpolation inequalities (1.1) are the recurrent tools used in [6] to improve the result established before by Scheffer concerning the dimension of the subset of singularities (see [31–34], see also [28] for streamlined proofs based on Scheffer’s arguments). The focus is specifically on inequalities of the form:

1.12 $$\begin{aligned} \left( \int _{{\mathbb {R}}^3} \tau ^{\gamma r} |u|^{r} \textrm{d}x \right) ^{1 / r}\leqslant &   C \left( \int _{{\mathbb {R}}^3} | x |^{2 \alpha } \left| \nabla u \right| ^2 \textrm{d}x \right) ^{a / 2}\nonumber \\  &   \left( \int _{{\mathbb {R}}^3} \tau ^{2 \beta } |u|^2 \textrm{d}x \right) ^{(1 - a) / 2} \quad \forall u \in C^{\infty }_c ( {\mathbb {R}}^3), \end{aligned}$$

corresponding to the case $p= q= 2$ and $N= 3$ in (1.1), where the weight is $\tau (x) = | x |$ in some cases and $\tau (x) = (\varepsilon + | x |^2)^{1 / 2}$ in others. The authors say that they will not attempt to present the most general class of inequalities in the form (1.1) because the weighted interpolation inequalities they need are all of the specific form (1.12). Rather, they refer the reader to [7], where a systematic analysis is performed, and to [35] for similar results involving derivatives of higher order. We note in passing that there is no treatment of weights other than $\tau (x) = | x |$ in [7] and, therefore, no mention concerning the weight $\tau (x) = (\varepsilon + | x |^2)^{1 / 2}$ in [7]. Notably, the weight $\tau (x) = (\varepsilon + | x |^2)^{1 / 2}$ is not treated in [7], though it is within the scope of the present paper (cf. (2.3)).

The profound influence of CKN inequalities is evident across various mathematical fields, such as partial differential equations, geometric analysis, and mathematical physics. These inequalities have proven instrumental in the analysis of singular nonlinear elliptic equations in divergence form. In  [3], the authors establish existence and nonexistence results for positive solutions of the elliptic equation:

$$\begin{aligned} - {{\textrm{div}}} \left( |x|^{\alpha } | \nabla u|^{p- 2} \nabla u \right) = |x|^{\beta } u^{p(\alpha , \beta ) - 1}, \end{aligned}$$

on a domain $\Omega \subseteq {\mathbb {R}}^{N}$, $N\geqslant 3$, subject to homogeneous Dirichlet boundary conditions. Their analysis accommodates potentially degenerate or singular cases: $1< p< N$, and $\alpha , \beta \in {\mathbb {R}}$ such that $p(\alpha , \beta ) :=p(N+ \beta ) / \left( N- p+ \alpha \right) > p$. A similar methodology is employed in [5] to investigate the existence of multiple solutions for nonhomogeneous singular elliptic equations involving critical CKN exponents. Further insights into applications of the Caffarelli–Kohn–Nirenberg inequalities in elliptic problems can be found in [1, 2, 9, 19]. It is also worth noting the complementary findings in [29, 30], which extensively explore continuous embeddings of Sobolev spaces with power-type weights, which offer valuable insights into the functional framework underlying many applications of CKN inequalities to elliptic problems. Earlier results on this topic can be found in [18, 38], and we refer to [24] for an accessible and consistent exposition of the subject.

Another extensive area of research focuses on analyzing CKN inequalities within specific parameter ranges to determine the best constants, the existence of extremal functions, and their qualitative properties. The relevant literature traces back to contributions in [8], which follow a lineage of work initiated in [37] for Sobolev inequalities and in [27] for Hardy–Littlewood–Sobolev inequalities. An interesting finding emerging from these studies is that, unlike Gagliardo-Nirenberg inequalities, there is a phenomenon of symmetry breaking in the CKN inequalities, i.e., minimizers of such inequalities may not be radially symmetric functions when posed in the whole space or in balls. This symmetry breaking, where optimal constants are achieved by non-radial functions, has been rigorously established in [16, 17] for certain regions of the parameter space. Conversely, in other regions, extremal functions retain radial symmetry and are monotonically decreasing. Additionally, quantitative stability results have been presented in [39], generalizing previous work on the sharp stability of profile decompositions for the Sobolev inequality under small perturbations [21].

Finally, it is worth mentioning the rigidity result in [40], which shows that Euclidean space is the only complete Riemannian manifold with non-negative Ricci curvature that satisfies specific CKN inequalities. The parameter range examined includes the classical Nash inequality, thereby addressing an open question posed by Ledoux in [26].

### Outline

The remainder of this paper is organized as follows. Section 2 begins with an introductory preamble that sets the stage for the research presented here. We then introduce our main results findings, starting with a generalized integration by parts formula (cf. Theorem 1), which encompasses a broader class of weights, an extended range of exponents, and accounts for potential regularity loss at the boundary. In the subsequent Corollary 1, we demonstrate that the classical radial integration by parts formula is a limiting case of our generalized formula. Subsequently, inspired by the ideas of [7], we present several new results on Caffarelli–Kohn–Nirenberg (CKN) inequalities that extend the original results of [7]. Each theorem is accompanied by a corollary, illustrating how our generalized CKN inequalities reduce to the classical ones as special cases. Additionally, we explore new findings related to CKN inequalities for functions in $C^{\infty }( {\overline{\Omega }})$. Our exploration does not cover the full range of parameters addressed in [7]; instead, we focus on the regimes where the radial integration by parts formula is central to the analysis. In presenting these results, we have aimed for clarity and readability, highlighting methodological patterns applicable across different contexts. All proofs and detailed derivations are provided in Sect. 3.

## Contributions of the present work

In their seminal paper [7], Caffarelli, Kohn, and Nirenberg made constant use of what they call *radial integration by parts*, which for any $u \in C_c^{\infty }( {\mathbb {R}}^{N})$, $N\geqslant 1$, reads as

2.1 $$\begin{aligned} \int _{{\mathbb {R}}^{N}} | x |^{\theta } | u (x) |^{r} \textrm{d}x \leqslant C \int _{{\mathbb {R}}^{N}} | x |^{\theta + 1} | u (x) |^{r- 1} \left| \nabla u (x) \right| \textrm{d}x \quad \forall u \in C^{\infty }_c ( {\mathbb {R}}^{N} ), \end{aligned}$$

provided $r> 1$ (see [7, Eq. (2.13) and note that $0< a < 1$ in there]), $\theta > - 1$ (see [7, Eq. after (3.15)]), which entails $N+ \theta > 0$, and for some constant $C > 0$ independent of *u*. The radial integration by parts formula (2.1) is the crucial simplification used to obtain the weighted interpolation inequalities (1.1) whose derivation under general hypotheses on the involved parameters is the primary goal of [7].

The name radial integration by parts comes from the idea behind it, which consists of integrating in polar coordinates to obtain that

2.2 $$\begin{aligned}&\int _{{\mathbb {R}}^N} | x |^{\theta } | u (x) |^{r} \textrm{d}x \nonumber \\&\quad = \frac{1}{N+ \theta } \int _0^{+ \infty } \partial _s \left( s^{N+ \theta } \right) \int _{\xi \in {\mathbb {S}}^{N - 1}} | u (s \xi ) |^{r} \textrm{d}\xi \textrm{d}s \nonumber \\&\quad = - \frac{r}{N+ \theta } \int _0^{+ \infty } s^{N- 1} s^{\theta } \int _{\xi \in {\mathbb {S}}^{N - 1}} \operatorname {sign} (u (s \xi )) {| u (s \xi ) |^{r- 1}} \nabla u (s \xi ) \cdot (s \xi ) \textrm{d}\xi \textrm{d}s \nonumber \\&\quad = - \frac{r}{N+ \theta } \int _{{\mathbb {R}}^N} | x |^{\theta } \operatorname {sign} (u (x)) {| u (x) |^{r- 1}} \nabla u (x) \cdot x \textrm{d}x. \end{aligned}$$

Hence, formula (2.1) holds with $C :=r/ \left( N+ \theta \right)$. Clearly, formula (2.1) still holds when $u \in C_c^{\infty } ( \Omega )$ where $\Omega$ is an arbitrary open subset of ${\mathbb {R}}^{N}$. However, it does not hold anymore when $\Omega$ is a smooth bounded domain and $u \in C^{\infty } ( {\overline{\Omega }})$, due to the potential introduction of boundary singularities during integration by parts in polar coordinates: if $u \in C^{\infty } ( {\overline{\Omega }})$, integrating by parts in polar coordinates can introduce singularities for those values of $s \in {\mathbb {R}}_+$ such that $\partial \Omega \cap s {\mathbb {S}}^{N - 1} \ne \emptyset$. Furthermore, the argument that leads to (2.2) fails when $0 < r\leqslant 1$ because of the loss of regularity in the function $\left| \, \cdot \, \right| ^{r}$.

The objectives of this paper are twofold: first, to extend the classical radial integration by parts formula (2.1) to encompass more general weight functions and function classes, and second, to show how this generalized formula can be used to obtain certain generalizations of the CKN inequalities, in the very same spirit (2.1) served to obtain the family of CKN inequalities (1.1). Our generalization of (2.1) accommodates more general exponents ($r> 0$), a broader family of weights, and even functions that are $C_c^{\infty } ( {\overline{\Omega }})$ with $\Omega$ bounded, provided the boundary $\partial \Omega$ is sufficiently regular to allow for the use of the divergence theorem. The provided estimates also yield explicit upper bounds on the optimal constants, independent of the domain’s geometry, consistent with the scaling invariant nature of the inequalities.

### Statement of main results

Our analysis focuses on radial weights of the form

2.3 $$\begin{aligned} \omega (x) :=(\lambda + | x |^{\mu })^{\gamma / \mu }, \end{aligned}$$

with $\lambda> 0, \mu > 0, \gamma \ne 0$. While $\omega$ depends on the parameters $\lambda> 0, \mu > 0$, and $\gamma \ne 0$, we omit these dependencies in the notation for simplicity.

The family (2.3) recovers the classical radial weight $\omega (x) = | x |^{\gamma }$ in the limit as $\lambda \rightarrow 0$. The validity of all the forthcoming estimates in the case $\lambda = 0$ will essentially be established by applying the dominated convergence theorem and the monotone convergence theorem from integral calculus.

The following constant will be recurrently used throughout the paper:

2.4 $$\begin{aligned} \kappa _+ :=\left\{ \begin{array}{lll} N&  \text {if} &  \gamma > 0,\\ N+ \gamma r&  \text {if} &  \gamma < 0. \end{array} \right. \end{aligned}$$

It should be noted that $\kappa _+$ is not necessarily positive when $\gamma < 0$, the condition $\kappa _+ > 0$ being equivalent to the condition $N+ \gamma r> 0$. As assumed to establish (1.1) in [7] (cf. (1.3)), also our results are significative only when $N+ \gamma r> 0$; the motivation for the importance of the condition $N+ \gamma r> 0$ is explained by Lemma 1.

The initial result we present is a generalized radial integration by parts formula.

#### [Style2 Style3 Style3]Theorem 1

Let $\Omega \subseteq {\mathbb {R}}^{N}$ be an open set and $r> 0$. For $\lambda> 0, \mu > 0, \gamma \ne 0$, consider the weight $\omega$ defined by (2.3) and let $\kappa _+$ be the constant defined by (2.4). The following inequalities hold:

i. If $\Omega \subseteq {\mathbb {R}}^{N}$ is a bounded open set with a Lipschitz boundary, then for every $u \in C^{\infty }({\overline{\Omega }})$, there holds
  2.5 $$\begin{aligned} \kappa _+ \int _{\Omega } \omega ^{r} (x) | u (x) |^{r} \textrm{d}x&\leqslant r\int _{\Omega } | x | \omega ^{r} (x) | u (x) |^{r- 1} \left| \nabla u (x) \right| \textrm{d}x \nonumber \\&\quad + \int _{\partial \Omega } \omega ^{r} (\xi ) | u (\xi ) |^{r} (\xi \cdot \varvec{\nu } (\xi )) \textrm{d}\xi , \end{aligned}$$
  provided that $\kappa _+ > 0$, i.e., that $\gamma$ is such that $N+ \gamma r> 0$. Here $\varvec{\nu }$ is the outward-pointing unit normal to $\partial \Omega$.
ii. If $\Omega \subseteq {\mathbb {R}}^{N}$ is an arbitrary open set, then for every $u \in C^{\infty }_c ( \Omega )$ there holds
  2.6 $$\begin{aligned} \kappa _+ \int _{\Omega } \omega ^{r} (x) | u (x) |^{r} \textrm{d}x&\leqslant r\int _{\Omega } | x | \omega ^{r} (x) | u (x) |^{r- 1} \left| \nabla u (x) \right| \textrm{d}x, \end{aligned}$$
  provided that $\kappa _+ > 0$, i.e., that $\gamma$ is such that $N+ \gamma r> 0$.

#### Remark

When $0< r< 1$, the first terms on the right-hand sides of (2.5) and (2.6) are of the form $\left| \nabla u \right| / | u |^{1 - r}$, which appears undefined at locations where *u* vanishes. Nonetheless, as will be elucidated in the proof of Theorem 1, the correct interpretation of the right-hand sides of (2.5) and (2.6) when $0< r< 1$ is that $\left| \nabla u \right| / | u |^{1 - r} = 0$ at points where $| u | = 0$.

#### Remark

Note that when $\gamma > 0$, the condition $\kappa _+ > 0$ is always satisfied.

A limiting case of our radial integration by parts formula, precisely the case where $\lambda \rightarrow 0$ in the weight (2.3), gives the classical formula (2.1), which Caffarelli, Kohn, and Nirenberg recurrently use to prove the first-order interpolation inequalities in [7].

#### Corollary 1

Let $\Omega \subseteq {\mathbb {R}}^{N}$ be an open set. Let $r> 0$, $\theta \in {\mathbb {R}}$, and define $\kappa _{\theta } :=\left( N+ \theta \right)$ if $\theta < 0$ and $\kappa _{\theta } = N$ if $\theta > 0$. The following inequalities hold:

i. If $\Omega \subseteq {\mathbb {R}}^{N}$ is a bounded open set with a Lipschitz boundary, then for every $u \in C^{\infty } ( {\overline{\Omega }})$ there holds
  2.7 $$\begin{aligned} \kappa _{\theta } \int _{\Omega } | x |^{\theta } | u (x) |^{r} \textrm{d}x&\leqslant r\int _{\Omega } | x |^{\theta + 1} | u (x) |^{r- 1} \left| \nabla u (x) \right| \textrm{d}x \nonumber \\&\quad + \int _{\partial \Omega } | x |^{\theta } | u (\xi ) |^{r} (\xi \cdot \varvec{\nu } (\xi )) \textrm{d}\xi . \end{aligned}$$
  provided that $\kappa _+ > 0$, i.e., that $N+ \theta > 0$ if $\theta < 0$. Here $\varvec{\nu }$ is the outward-pointing unit normal to $\partial \Omega$.
ii. If $\Omega \subseteq {\mathbb {R}}^{N}$ is an arbitrary open set, then for every $u \in C^{\infty }_c ( \Omega )$ there holds
  2.8 $$\begin{aligned} \kappa _{\theta } \int _{\Omega } | x |^{\theta } | u (x) |^{r} \textrm{d}x&\leqslant r\int _{\Omega } | x |^{\theta + 1} | u (x) |^{r- 1} \left| \nabla u (x) \right| \textrm{d}x, \end{aligned}$$
  provided that $\kappa _{\theta } > 0$, i.e., that $N+ \theta > 0$ if $\theta < 0$.

#### Remark

When $0< r< 1$, the correct understanding of the right-hand sides of (2.7) and (2.8) is that $\left| \nabla u \right| / | u |^{1 - r} = 0$ at points where $| u | = 0$. This is evident from the proof of Theorem 1.

Having introduced our radial integration by parts formula, we can focus on its consequences to obtain generalized CKN-type inequalities. In presenting our results, we tried to promote clearness and readability and emphasize the methodological pattern that can be used in different circumstances. In fact, a similar approach was already fruitfully applied by the authors in [13] to obtain a unified point of view on Hardy–Poincaré inequalities in classical and variable Sobolev spaces, in [15] to revisit Korn and Poincaré-Korn inequalities from a different perspective, and even in [14] to investigate global minimizers of a perturbed Dirichlet energy subject to a boundary penalization.

In extending the CKN inequalities to our setting, we do not treat all range of parameters covered in [7]; we focus on those regimes where radial integration by parts formula is the key step. The first result in that direction is the following one.

#### [Style2 Style3 Style3]Theorem 2

Let $\Omega \subseteq {\mathbb {R}}^{N}$, $N\geqslant 1$, be an open set *(*bounded or not*)*. Let $\lambda , \mu > 0, \gamma \ne 0$ be the parameters in the radial weight $\omega$ defined by (2.3) and let $\kappa _+$ be the constant defined by (2.4). Assume that the parameters $a, p, q, r> 0$ are such that

2.9 $$\begin{aligned} 1 \geqslant a> \max \left( \frac{1}{r}, \frac{1}{q+ 1} \right) , \quad \kappa _+ > 0, \end{aligned}$$

with the condition $\kappa _+ > 0$ being relevant only when $\gamma < 0$ and being equivalent to $N+ r\gamma > 0$.

Then, for any $\alpha , \beta \in {\mathbb {R}}$, the following inequality holds

2.10 $$\begin{aligned} \left\| \omega | u | \right\| _{L^{r} ( \Omega )} \leqslant \left( \frac{r}{\kappa _+} \right) ^a \left\| \, | x |^{\alpha } \omega ^{\alpha / a} \left| \nabla u \right| \, \right\| _{L^{p} ( \Omega )}^a \; \cdot \; \left\| \, | x |^{\beta } \omega ^{\beta / a} | u | \, \right\| _{L^{q} ( \Omega )}^{1 - a} \quad \forall u \in C^{\infty }_c ( \Omega ), \end{aligned}$$

whenever the involved parameters satisfy the following relations:

2.11 $$\begin{aligned} \alpha + \beta \left( \frac{1}{a} - 1 \right) =1, \qquad \frac{1}{p} + \frac{a^{- 1} - 1}{q} =\frac{1}{a r} . \end{aligned}$$

#### Remark

In more explicit terms, inequality (2.10) reads as

2.12 $$\begin{aligned} \left( \int _{\Omega } (\lambda + | x |^{\mu })^{r\gamma / \mu } |u|^{r} \textrm{d}x \right) ^{1 / r}&\leqslant \left( \frac{r}{\kappa _+} \right) ^a \left( \int _{\Omega } | x |^{\alpha p} (\lambda + | x |^{\mu })^{\frac{\alpha p\gamma }{\mu a}} \left| \nabla u \right| ^{p} \textrm{d}x \right) ^{a / p} \nonumber \\&\quad \cdot \left( \int _{\Omega } | x |^{\beta q} (\lambda + | x |^{\mu })^{\frac{\beta q\gamma }{\mu a}} |u|^{q} \textrm{d}x \right) ^{(1 - a) / q} . \end{aligned}$$

As a limiting case $(\lambda \rightarrow 0)$ of the previous interpolation inequality, we get the CKN of [7] in the regime (2.9).

#### Corollary 2

Let $\Omega \subseteq {\mathbb {R}}^{N}$, $N\geqslant 1$, be an open set *(*bounded or not*)*. Let $\gamma \ne 0$ and let $\kappa _+$ be the constant defined by (2.4). Assume that $a, p, q, r> 0$ are such that

2.13 $$\begin{aligned} 1 \geqslant a> \max \left( \frac{1}{r}, \frac{1}{q+ 1} \right) , \quad \quad \kappa _+ > 0, \end{aligned}$$

with the condition $\kappa _+ > 0$ being relevant only when $\gamma < 0$ and equivalent to $N+ r\gamma > 0$.

Then, for any $\alpha , \beta \in {\mathbb {R}}$, the following inequality holds

2.14 $$\begin{aligned} \Vert | x |^{\gamma } | u | \Vert _{L^{r} ( \Omega )} \leqslant \left( \frac{r}{\kappa _+} \right) ^a \left\| \, | x |^{\alpha } \left| \nabla u \right| \, \right\| _{L^{p} ( \Omega )}^a \; \cdot \; \left\| \, | x |^{\beta } | u | \, \right\| _{L^{q} ( \Omega )}^{1 - a} \quad \forall u \in C^{\infty }_c ( \Omega ), \end{aligned}$$

whenever the involved parameters satisfy the following relations:

2.15 $$\begin{aligned} \alpha + \beta \left( \frac{1}{a} - 1 \right) =1 + \frac{\gamma }{a}, \qquad \frac{1}{p} + \frac{a^{- 1} - 1}{q} =\frac{1}{a r} . \end{aligned}$$

#### Remark

To facilitate a comparative analysis between our parameter set (2.15) and that presented in [7], it is beneficial to reformulate equation (2.15) as follows:

$$\begin{aligned} a \left( \frac{\alpha - 1}{N} \right) + (1 - a) \frac{\beta }{N} =\frac{\gamma }{N}, \quad \frac{a}{p} + \frac{1 - a}{q} =\frac{1}{r}. \end{aligned}$$

This reformulation clearly demonstrates that the parameters adhere to the dimensional balance equation (1.4), i.e., they satisfy the relation

$$\begin{aligned} a \left( \frac{\alpha - 1}{N} \right) + (1 - a) \frac{\beta }{N} + \frac{a}{p} + \frac{1 - a}{q} = \frac{\gamma }{N} + \frac{1}{r}. \end{aligned}$$

Contrary to the approach taken in [7], where conditions (1.2) and (1.3) are assumed as prerequisites, our analysis reveals that not all those assumptions are necessary. For instance, under the condition $a = 1$, our parameter conditions (2.15) imply that the interpolation inequality (2.14) holds when

$$\begin{aligned} \gamma = \alpha - 1, \quad p= r\end{aligned}$$

and $p$ and $q$ are constrained by the conditions delineated in (2.13), namely, $p\geqslant 1$ and $q > 0$. Notably, [7] imposes the more stringent requirement $q\geqslant 1$ from the outset.

Similarly, for $a = 3 / 4$, our parameter conditions (2.15) imply that the interpolation inequality (2.14) holds when

$$\begin{aligned} \gamma = \frac{\beta - 3 (1 - \alpha )}{4}, \quad \frac{1}{r} = \frac{3 / p+ 1 / q}{4}, \end{aligned}$$

and $r$ and $q$ are constrained by the conditions delineated in (2.13), which, assuming for simplicity $p > 1$, lead to the condition

$$\begin{aligned} q > \frac{1}{3} \left( \frac{p}{p- 1} \right) . \end{aligned}$$

Again, [7] imposes the more stringent requirements $q\geqslant 1$ from the outset.

#### Remark

Note that the equation (2.11) that gives the parameters $\alpha$ and $\beta$ under which (2.10) holds depends only on *a*. Now, instead, the relation (2.15) that gives the parameters $\alpha$ and $\beta$ depends also on the exponent $\gamma$ of the standard radial weight $| x |^{\gamma }$.

#### Remark

In more explicit terms, inequality (2.14) reads as

2.16 $$\begin{aligned} \left( \int _{\Omega } | x |^{r\gamma } |u|^{r} \textrm{d}x \right) ^{1 / r}&\leqslant \left( \frac{r}{\kappa _+} \right) ^a \left( \int _{\Omega } | x |^{\alpha p} \left| \nabla u \right| ^{p} \textrm{d}x \right) ^{a / p} \nonumber \\&\quad \cdot \left( \int _{\Omega } | x |^{\beta q} |u|^{q} \textrm{d}x \right) ^{(1 - a) / q} . \end{aligned}$$

#### [Style2 Style3 Style3]Theorem 3

Let $\Omega \subseteq {\mathbb {R}}^{N}$, $N\geqslant 1$, be an open set *(*bounded or not*)*. Let $\lambda , \mu > 0, \gamma \ne 0$ be the parameters in the radial weight $\omega$ defined by (2.3) and let $\kappa _+$ be the constant defined by (2.4). Assume that $a, p, q, r> 0$ are such that

2.17 $$\begin{aligned} 1 \geqslant a = 1 / r, \quad \quad \kappa _+ > 0, \end{aligned}$$

with the condition $\kappa _+ > 0$ being relevant only when $\gamma < 0$ and equivalent to $N+ r\gamma > 0$.

Then, for any $\alpha , \beta \in {\mathbb {R}}$, the following interpolation inequality holds

2.18 $$\begin{aligned} \left\| \omega | u | \right\| _{L^{r} ( \Omega )} \leqslant \left( \frac{r}{\kappa _+} \right) ^{1 / r} \left\| \, | x |^{\alpha } \omega ^{\alpha r} \left| \nabla u \right| \, \right\| _{L^{p}( \Omega )}^{1 / r} \; \cdot \; \left\| \, | x |^{\beta } \omega ^{\beta r} | u | \, \right\| _{L^{q} ( \Omega )}^{1 - 1 / r} \quad \forall u \in C^{\infty }_c ( \Omega ), \end{aligned}$$

holds whenever the involved parameters are solutions of the following equations:

2.19 $$\begin{aligned} \alpha + \beta \left( r- 1 \right) =1, \qquad \frac{1}{p} + \frac{r- 1}{q} =1. \end{aligned}$$

#### Remark

Formally, inequality (2.18) appears to be a limiting case of (2.10), in the sense that it is nothing but (2.10) with the formal substitution $a :=1 / r$. Indeed, the constraint (2.11) for $a = 1 / r$ reduces to $\frac{1}{p} + \frac{r- 1}{q} =1$. However, in the proof of the case $a :=1 / r$, we rely on the classical (two-factors) Hölder inequality.

#### Corollary 3

Let $\Omega \subseteq {\mathbb {R}}^{N}$, $N\geqslant 1$, be an open set *(*bounded or not*)*. Let $\gamma \ne 0$ and let $\kappa _+$ be the constant defined by (2.4). Assume that $a, p, q, r> 0$ are such that

2.20 $$\begin{aligned} 1 \geqslant a = 1 / r, \quad \quad \kappa _+ > 0, \end{aligned}$$

with the condition $\kappa _+ > 0$ being relevant only when $\gamma < 0$ and equivalent to $N+ r\gamma > 0$.

Then, for any $\alpha , \beta \in {\mathbb {R}}$, the following interpolation inequality holds

2.21 $$\begin{aligned} \Vert | x |^{\gamma } | u | \Vert _{L^{r} ( \Omega )} \leqslant \left( \frac{r}{\kappa _+} \right) ^{1 / r} \left\| \, | x |^{\alpha } \left| \nabla u \right| \, \right\| _{L^{p} ( \Omega )}^{1 / r} \; \cdot \; \left\| \, | x |^{\beta } | u | \, \right\| _{L^{q} \left( \Omega \right) }^{1 - 1 / r}, \quad \forall u \in C^{\infty }_c ( \Omega ), \end{aligned}$$

holds whenever the involved parameters are solutions of the following equations:

2.22 $$\begin{aligned} \alpha + \beta \left( r- 1 \right) =1 + \gamma r, \qquad \frac{1}{p} + \frac{r- 1}{q} =1. \end{aligned}$$

#### Remark

Note that the equation (2.19) that gives the parameters $\alpha$ and $\beta$ for which (2.18) holds depends only on *a*. Now, instead, the relation (2.15) that gives the parameters $\alpha$ and $\beta$ depends also on the exponent $\gamma$ of the standard radial weight $| x |^{\gamma }$.

#### Remark

To compare our range of parameters (2.19) with the one provided in [7], it is convenient to reformulate equation (2.19) as follows:

$$\begin{aligned} a \left( \frac{\alpha - 1}{N} \right) + (1 - a) \frac{\beta }{N} = \frac{\gamma }{N}, \quad a \frac{1}{p} + (1 - a) \frac{1}{q} =\frac{1}{r}, \quad a = \frac{1}{r}. \end{aligned}$$

It becomes immediately apparent that the parameters involved satisfy the dimensional balance equation (1.4). Unlike [7], where assumptions (1.2) and (1.3) are imposed, our approach does not necessitate these conditions. For instance, by setting $a =r=1$, our conditions (2.17) and (2.22) imply that the interpolation inequality (2.14) is satisfied when

$$\begin{aligned} \gamma =\alpha - 1, \quad p=r=1, \end{aligned}$$

with, again, the condition $q > 0$ being permissible. In contrast, [7] assumes $q\geqslant 1$ from the very beginning.

Our last contribution shows how the CKN inequalities established so far can easily be extended to functions that do not vanish on the boundary. The proofs will rely on the radial integration by parts formula with a boundary remainder term.

#### [Style2 Style3 Style3]Theorem 4

Let $\Omega \subseteq {\mathbb {R}}^{N}$, $N\geqslant 1$, be a bounded open set with a Lipschitz boundary. Let $\lambda , \mu > 0, \gamma \ne 0$ be the parameters in the radial weight $\omega$ defined by (2.3) and let $\kappa _+$ be the constant defined by (2.4). Assume that $a, p, q, r> 0$ are such that

2.23 $$\begin{aligned} 1 \geqslant a = 1 / r, \quad \quad \kappa _+ > 0. \end{aligned}$$

with the condition $\kappa _+ > 0$ being relevant only when $\gamma < 0$ and equivalent to $N+ r\gamma > 0$.

Then, for any $\alpha , \beta \in {\mathbb {R}}$ and every $u \in C^{\infty }( {\overline{\Omega }})$, the following interpolation inequality holds

2.24 $$\begin{aligned} \left\| \omega | u | \right\| _{L^{r} ( \Omega )}&\leqslant \left( \frac{2 r}{\kappa _+} \right) ^{1 / r} \left\| \, | x |^{\alpha } \omega ^{\alpha r} \left| \nabla u \right| \, \right\| _{L^{p} ( \Omega )}^{1 / r} \; \cdot \; \left\| \, | x |^{\beta } \omega ^{\beta r} | u | \, \right\| _{L^{q} ( \Omega )}^{1 - 1 / r} \nonumber \\&\quad + \left( \frac{2}{\kappa _+} \right) ^{1 / r} \left( \int _{\partial \Omega } \omega ^{r} | u |^{r} (\xi \cdot \varvec{\nu }) \textrm{d}\xi \right) ^{1 / r}, \end{aligned}$$

provided the involved parameters are solutions of the following equations:

2.25 $$\begin{aligned} \alpha + \beta \left( r- 1 \right) = 1, \qquad \frac{1}{p} + \frac{1}{q} =1. \end{aligned}$$

From Theorem 4, after observing that $\omega (x) = (\lambda + | x |^{\mu })^{\gamma / \mu } \rightarrow | x |^{\gamma }$ pointwise in ${\mathbb {R}}^{N}$ when $\lambda \rightarrow 0$, one can infer the following family of CKN inequalities with boundary term as remainder.

#### Corollary 4

Let $\Omega \subseteq {\mathbb {R}}^{N}$, $N\geqslant 1$, be a bounded open set with a Lipschitz boundary. Let $\gamma \ne 0$ and let $\kappa _+$ be the constant defined by (2.4). Assume that $a, p, q, r> 0$ are such that

2.26 $$\begin{aligned} 1 \geqslant a = 1 / r, \quad \quad \kappa _+ > 0. \end{aligned}$$

with the condition $\kappa _+ > 0$ being relevant only when $\gamma < 0$ and equivalent to $N+ r\gamma > 0$.

Then, for any $\alpha , \beta \in {\mathbb {R}}$ and for every $u \in C^{\infty }( {\overline{\Omega }})$ the following inequality holds

2.27 $$\begin{aligned} \Vert | x |^{\gamma } | u | \Vert _{L^{r} ( \Omega )}&\leqslant \left( \frac{2 r}{\kappa _+} \right) ^{1 / r} \left\| \, | x |^{\alpha } \left| \nabla u \right| \, \right\| _{L^{p} ( \Omega )}^{1 / r} \; \; \cdot \; \left\| \, | x |^{\beta } | u | \, \right\| _{L^{q} ( \Omega )}^{1 - 1 / r} \nonumber \\&\quad + \left( \frac{2}{\kappa _+} \right) ^{1 / r} \left( \int _{\partial \Omega } | x |^{\gamma r} | u |^{r} (\xi \cdot \varvec{\nu }) \textrm{d}\xi \right) ^{1 / r}, \end{aligned}$$

provided the involved parameters are solutions of the following equations:

2.28 $$\begin{aligned} \alpha + \beta \left( r- 1 \right) = 1 + \gamma r, \qquad \frac{1}{p} + \frac{1}{q} =1. \end{aligned}$$

## Radial integration by parts formula and CKN-type inequalities: proofs

In the arguments that follow, we will make constant use of the following observation.

### Lemma 1

Let $r> 0$. For $\lambda> 0, \mu > 0, \gamma \ne 0$, consider the weight $\omega (x) :=(\lambda + | x |^{\mu })^{\gamma / \mu }$ and set $\kappa _+ :=N+ \gamma r$ if $\gamma < 0$ and $\kappa _+ :=N$ if $\gamma > 0$. The following estimate holds:

3.1 $$\begin{aligned} {\textrm{div}}_x \left[ \omega ^{r} x \right] \geqslant \; \kappa _+ \omega ^{r} (x) \end{aligned}$$

Note that, $\kappa _+$ is a positive constant if, and only if, $N+ \gamma r> 0$.

### Proof of Lemma 1

It is a matter of verification by computation. We have

$$\begin{aligned} {\textrm{div}}_x \left[ \omega ^{r} x \right]&=\nabla _x \left[ \omega ^{r} \right] \cdot x + N\omega ^{r} \\&=r\omega ^{r- 1} \nabla _x \left[ \omega \right] \cdot x + N\omega ^{r} \\&=r\omega ^{r- 1} \frac{\gamma }{\mu } (\lambda + | x |^{\mu })^{(\gamma / \mu ) - 1} \mu | x |^{\mu - 1} \frac{x}{| x |} \cdot x + N\omega ^{r} \\&=r\omega ^{r- 1} \gamma (\lambda + | x |^{\mu })^{(\gamma / \mu )} \frac{| x |^{\mu }}{(\lambda + | x |^{\mu })} + N\omega ^{r} \\&=\gamma r\omega ^{r} \frac{| x |^{\mu }}{(\lambda + | x |^{\mu })} + N\omega ^{r} \\&=\left( N+ \gamma r\frac{| x |^{\mu }}{\lambda + | x |^{\mu }} \right) \omega ^{r} (x) \\&\geqslant \; \kappa _+ \omega ^{r} (x), \end{aligned}$$

from which (3.1) follows. Note that, by definition, if $\gamma > 0$ then $\kappa _+ > 0$, while if $\gamma < 0$ then $\kappa _+ > 0$ provided that $N+ \gamma r> 0$. Overall, we get that $\kappa _+ > 0$ if, and only if, $N+ \gamma r> 0$. $\square$

### Proof of Theorem 1

For every $\varepsilon > 0$, we set $\varvec{u}_{\varepsilon } :=(u, \varepsilon )$ and $| \varvec{u}_{\varepsilon } |^2 = u^2 + \varepsilon ^2$, so that for every $r> 0$, $| \varvec{u}_{\varepsilon } |^{r} \in C^{\infty }( {\overline{\Omega }})$ and

$$\begin{aligned} \nabla | \varvec{u}_{\varepsilon } |^{r} = \nabla [ ( u^2 {+ \varepsilon ^2} )^{\frac{r}{2}} ] = \frac{r}{2} ( u^2 {+ \varepsilon ^2} )^{\frac{r}{2} - 1} 2 u \nabla u = r| \varvec{u}_{\varepsilon } |^{r- 2} u \nabla u. \end{aligned}$$

We preliminary observe that by Lemma 1, pointwise in $\Omega$, there holds

3.2 $$\begin{aligned} {\textrm{div}}_x \left[ | \varvec{u}_{\varepsilon } |^{r} \omega ^{r} x \right]&=| \varvec{u}_{\varepsilon } |^{r} {\textrm{div}}_x \left[ \omega ^{r} x \right] + r\omega ^{r} | \varvec{u}_{\varepsilon } |^{r- 2} u \nabla u \cdot x \nonumber \\&\geqslant \kappa _+ \omega ^{r} | \varvec{u}_{\varepsilon } |^{r} + r\omega ^{r} | \varvec{u}_{\varepsilon } |^{r- 1} \frac{u}{| \varvec{u}_{\varepsilon } |} \nabla u \cdot x. \end{aligned}$$

After that, we can focus on the proofs of items *i.* and *ii*.

#### Proof of i

By the divergence theorem, integrating over $\Omega$ relation (3.2) we obtain that

3.3 $$\begin{aligned} \kappa _+ \int _{\Omega } \omega ^{r} (x) | \varvec{u}_{\varepsilon } (x) |^{r} \textrm{d}x&\leqslant r\int _{\Omega } | x | \omega ^{r} (x) | \varvec{u}_{\varepsilon } (x) |^{r- 1} \left| \nabla u (x) \right| \textrm{d}x \nonumber \\&\quad + \int _{\partial \Omega } | \varvec{u}_{\varepsilon } (\xi ) |^{r} \omega ^{r} (\xi ) (\xi \cdot \varvec{\nu } (\xi )) \textrm{d}\xi . \end{aligned}$$

Next, if $r\geqslant 1$, then by dominated convergence, passing to the limit for $\varepsilon \rightarrow 0$, we get (2.5). In particular, for $r= 1$, we obtain that

$$\begin{aligned} \kappa _+ \int _{\Omega } \omega (x) | u (x) | \textrm{d}x \leqslant \int _{\Omega } | x | \omega (x) \left| \nabla u (x) \right| \textrm{d}x + \int _{\partial \Omega } | u (\xi ) | \omega (\xi ) (\xi \cdot \varvec{\nu } (\xi )) \textrm{d}\xi . \end{aligned}$$

Also, if $0< r< 1$, then by monotone convergence theorem

$$\begin{aligned} \lim _{\varepsilon \rightarrow 0} \int _{\Omega } | x | \omega ^{r} (x) | \varvec{u}_{\varepsilon } (x) |^{r- 1} \left| \nabla u (x) \right| \textrm{d}x = \int _{\Omega } | x | \omega ^{r} (x) \frac{\left| \nabla u (x) \right| }{| u (x) |^{1 - r}} \textrm{d}x, \end{aligned}$$

with the understanding that $\frac{\left| \nabla u (x) \right| }{| u (x) |^{1 - r}} = 0$ when $\left| \nabla u (x) \right| = 0$ and $u (x) = 0$. As before, passing to the limit for $\varepsilon \rightarrow 0$ in (3.3), we obtain that

$$\begin{aligned} \kappa _+ \int _{\Omega } \omega ^{r} (x) | u (x) |^{r} \textrm{d}x \leqslant r\int _{\Omega } | x | \omega ^{r} (x) \frac{\left| \nabla u (x) \right| }{| u (x) |^{1 - r}} \textrm{d}x + \int _{\partial \Omega } | u (\xi ) |^{r} \omega ^{r} (\xi ) (\xi \cdot \varvec{\nu } (\xi )) \textrm{d}\xi , \end{aligned}$$

which is nothing but (2.5). This concludes the proof of *i.* $\square$

#### Proof of ii

If $\Omega$ is a generic open set and $u \in C^{\infty }_c ( \Omega )$, then by the divergence theorem, integrating over $\Omega$ relation (3.2) we obtain that

3.4 $$\begin{aligned} \kappa _+ \int _{\Omega } \omega ^{r} (x) | \varvec{u}_{\varepsilon } (x) |^{r} \textrm{d}x&\leqslant r\int _{\Omega } | x | \omega ^{r} (x) | \varvec{u}_{\varepsilon } (x) |^{r- 1} \left| \nabla u (x) \right| \textrm{d}x \nonumber \\&\quad + \varepsilon ^{r} \int _{\partial \Omega } \omega ^{r} (\xi ) (\xi \cdot \varvec{\nu } (\xi )) \textrm{d}\xi . \end{aligned}$$

The only difference with (3.3) is in the boundary term, which now converges to zero as $\varepsilon ^{r}$ when $\varepsilon \rightarrow 0$. But then, arguing as before for the remaining terms, we get (2.6). This concludes the proof of *ii.* $\square$

### Proof of Corollary 1

Let us start with the radial integration by parts formula (2.5): for any $u \in C^{\infty } ( {\overline{\Omega }})$ there holds

3.5 $$\begin{aligned} \kappa _+ \int _{\Omega } \omega ^{r} (x) | u (x) |^{r} \textrm{d}x&\leqslant r\int _{\Omega } | x | \omega ^{r} (x) | u (x) |^{r- 1} \left| \nabla u (x) \right| \textrm{d}x \nonumber \\&\quad + \int _{\partial \Omega } \omega ^{r} (\xi ) | u (\xi ) |^{r} (\xi \cdot \varvec{\nu } (\xi )) \textrm{d}\xi . \end{aligned}$$

Note that pointwise in ${\mathbb {R}}^{N}$ we have $\omega (x) = (\lambda + | x |^{\mu })^{\gamma / \mu } \rightarrow | x |^{\gamma }$ when $\lambda \rightarrow 0$. Hence, by monotone convergence theorem if $\gamma < 0$ and dominated convergence theorem if $\gamma > 0$, we get that

3.6 $$\begin{aligned} \kappa _+ \int _{\Omega } | x |^{\gamma r} | u (x) |^{r} \textrm{d}x&\leqslant r\int _{\Omega } | x |^{\gamma r+ 1} | u (x) |^{r- 1} \left| \nabla u (x) \right| \textrm{d}x \nonumber \\&\quad + \int _{\partial \Omega } | x |^{\gamma r} | u (\xi ) |^{r} (\xi \cdot \varvec{\nu } (\xi )) \textrm{d}\xi . \end{aligned}$$

provided $N+ \gamma r> 0$ if $\gamma < 0$. Here $\kappa _+$ is the constant defined by (2.4). The formal assignment $\theta :=\gamma r$ gives (2.7) with $\kappa _{\theta }$ replacing $\kappa _+$. The same argument gives (2.8) from (2.6). $\square$

### Proof of Theorem 2

By Theorem 1, we know that if $\Omega \subseteq {\mathbb {R}}^{N}$ is an arbitrary open set, then for every $u \in C^{\infty }_c ( \Omega )$ there holds

3.7 $$\begin{aligned} \int _{\Omega } \omega ^{r} (x) | u (x) |^{r} \textrm{d}x&\leqslant \frac{r}{\kappa _+} \int _{\Omega } | x | \omega ^{r} (x) | u (x) |^{r- 1} \left| \nabla u (x) \right| \textrm{d}x, \end{aligned}$$

provided that $N+ r\gamma > 0$ if $\gamma < 0$ and with $\kappa _+$ given by $\kappa _+ = \left( N+ \gamma r\right)$ if $\gamma < 0$ and $\kappa _+ = N$ if $\gamma > 0$. We introduce real parameters $\alpha , \beta \in {\mathbb {R}}$, $a \in {\mathbb {R}}{\setminus } \{ 0 \}$, and rewrite (3.7) under the form

3.8 $$\begin{aligned} \int _{\Omega } \omega ^{r} | u |^{r} \textrm{d}x&\leqslant \frac{r}{\kappa _+} \int _{\Omega } \left( | x | \omega ^{1 / a} \right) ^{\alpha } \cdot \left| \nabla u \right| \nonumber \\&\qquad \cdot \left( \left( | x | \omega ^{1 / a} \right) ^{\beta } | u | \right) ^{1 / a - 1} \left( \omega ^{r- 1 / a} | u |^{ r- 1 / a} \right) \textrm{d}x, \end{aligned}$$

which is equivalent to (3.7) as soon as we chose $a, \alpha , \beta$ such that

3.9 $$\begin{aligned} \alpha + \beta \left( \frac{1}{a} - 1 \right) = 1. \end{aligned}$$

Now, from Hölder inequality on three factors, we get that if $p, q, s\in (0, + \infty ]$ are such that

3.10 $$\begin{aligned} \frac{1}{p} + \frac{(1 / a - 1)}{q} + \frac{1}{s} = 1, \quad 0< a^{- 1} - 1 < q, \end{aligned}$$

then

3.11 $$\begin{aligned} \left\| \omega | u | \right\| _{L^{r} ( \Omega )}^{r}&\leqslant \frac{r}{\kappa _+} \left\| \, \left( | x | \omega ^{1 / a} \right) ^{\alpha } \cdot \left| \nabla u \right| \, \right\| _{L^{p} ( \Omega )} \; \cdot \; \left\| \left( \left( | x | \omega ^{1 / a} \right) ^{\beta } | u | \right) ^{1 / a - 1} \right\| _{L^{\frac{q}{(1 / a - 1 )}} ( \Omega )} \nonumber \\&\quad \cdot \; \left\| \omega ^{r- 1 / a} | u |^{ r- 1 / a} \right\| _{L^{s} \left( \Omega \right) } \nonumber \\&=\frac{r}{\kappa _+} \left\| \, | x |^{\alpha } \omega ^{\alpha / a} \left| \nabla u \right| \, \right\| _{L^{p} ( \Omega )} \; \cdot \; \left\| \, | x |^{\beta } \omega ^{\beta / a} | u | \, \right\| _{L^{q} ( \Omega )}^{(1 / a - 1)} \; \nonumber \\&\quad \cdot \; \left\| \omega ^{r- 1 / a} | u |^{ r- 1 / a} \, \right\| _{L^{s} ( \Omega )} . \end{aligned}$$

After that, we choose $s> 0$ so that $\left( r- 1 / a \right) s= r$. This uniquely identifies $s:=a r/ \left( a r- 1 \right)$, and since $s$ has to be strictly positive, it also imposes the constraint

3.12 $$\begin{aligned} a r> 1 . \end{aligned}$$

With this choice of $s$, the third factor on the right-hand side of (3.11) reads as

$$\begin{aligned} \big \Vert \omega ^{r- 1 / a} | u |^{ r- 1 / a} \big \Vert _{L^{s} ( \Omega )} =\left( \int _{\Omega } \omega ^{r} | u |^{r} \textrm{d}x \right) ^{\frac{1}{r} \left( r- 1 / a \right) } =\left\| \omega | u | \right\| _{L^{r} ( \Omega )}^{r- 1 / a}, \end{aligned}$$

and the constraints in (3.10) reduce to

3.13 $$\begin{aligned} \frac{1}{p} + \frac{(1 / a - 1)}{q} =\frac{1}{a r}, \quad 0< a^{- 1} - 1 < q. \end{aligned}$$

Therefore, assuming without loss of generality that $\left\| \omega | u | \right\| _{L^{r}} \ne 0$ and dividing both sides of (3.11) by $\left\| \omega | u | \right\| _{L^{r}}^{r- 1 / a}$, we get that

$$\begin{aligned} \left\| \omega | u | \right\| _{L^{r} ( \Omega )} \leqslant \left( \frac{r}{\kappa _+} \right) ^a \big \Vert \, | x |^{\alpha } \omega ^{\alpha / a} \left| \nabla u \right| \, \big \Vert _{L^{p} ( \Omega )}^a \; \cdot \; \big \Vert \, | x |^{\beta } \omega ^{\beta / a} | u | \, \big \Vert _{L^{q} ( \Omega )}^{1 - a}, \end{aligned}$$

provided that the parameters $a, \alpha , \beta , p, q, r$ satisfy the constraints (cf. (3.9), (3.12), and (3.13))

3.14 $$\begin{aligned} \alpha + \beta \left( \frac{1}{a} - 1 \right) =1, \quad \frac{1}{p} + \frac{(1 / a - 1)}{q} =\frac{1}{a r}, \quad 0< a^{- 1} - 1 < q, \quad a r> 1, \quad p> 0. \end{aligned}$$

This completes the proof. $\square$

### Proof of Corollary 2

Let us start with the first-order interpolation inequality (2.10):

$$\begin{aligned} \left\| \omega | u | \right\| _{L^{r} ( \Omega )} \leqslant \left( \frac{r}{\kappa _+} \right) ^a \left\| \, | x |^{\alpha } \omega ^{\alpha / a} \left| \nabla u \right| \, \right\| _{L^{p} ( \Omega )}^a \cdot \; \left\| \, | x |^{\beta } \omega ^{\beta / a} | u | \, \right\| _{L^{q} ( \Omega )}^{1 - a} \quad \forall u \in C^{\infty }_c ( \Omega ), \end{aligned}$$

and note that pointwise in ${\mathbb {R}}^{N}$ we have $\omega (x) = (\lambda + | x |^{\mu })^{\gamma / \mu } \rightarrow | x |^{\gamma }$ when $\lambda \rightarrow 0$. But then, using the monotone convergence theorem if $\gamma < 0$ and the dominated convergence theorem if $\gamma > 0$, we get that

$$\begin{aligned} \Vert | x |^{\gamma } | u | \Vert _{L^{r} ( \Omega )}\leqslant &   \left( \frac{r}{\kappa _+} \right) ^a \big \Vert \, | x |^{\alpha (1 + \gamma a^{- 1})} \left| \nabla u \big | \, \right\| _{L^{p} ( \Omega )}^a \;\\  &   \cdot \; \big \Vert \, | x |^{\beta (1 + \gamma a^{- 1})} | u | \, \big \Vert _{L^{q} ( \Omega )}^{1 - a} \quad \forall u \in C^{\infty }_c ( \Omega ). \end{aligned}$$

First, assume that $\gamma \ne -a$. Then, if we set ${\tilde{\alpha }} :=\alpha (1 + \gamma a^{- 1})$ and ${\tilde{\beta }} :=\beta (1 + \gamma a^{- 1})$ we get

$$\begin{aligned} \Vert | x |^{\gamma } | u | \Vert _{L^{r} ( \Omega )} \leqslant \left( \frac{r}{\kappa _+} \right) ^a \big \Vert \, | x |^{{\tilde{\alpha }}} | \nabla u | \, \big \Vert _{L^{p} ( \Omega )}^a \; \cdot \; \big \Vert \, | x |^{{\tilde{\beta }}} | u | \, \big \Vert _{L^{q} ( \Omega )}^{1 - a} \end{aligned}$$

with ${\tilde{\alpha }}$ and ${\tilde{\beta }}$ such that ${\tilde{\alpha }} + {\tilde{\beta }} (a^{- 1} - 1) =1 + \gamma a^{- 1}$. Renaming back ${\tilde{\alpha }}$ and ${\tilde{\beta }}$ with $\alpha$ and $\beta$, we get (2.14).

Next, consider the case $\gamma =-a$. Let $\alpha$ and $\beta$ be any parameters that satisfy the first condition in (2.15), namely,

$$\begin{aligned} \alpha + \beta \left( \frac{1}{a} - 1 \right) =0. \end{aligned}$$

In this situation, we apply the previously obtained inequality (2.14) with a perturbation: for any $\varepsilon >0$, we set

$$\begin{aligned} \gamma _\varepsilon :=-a+\varepsilon ,\quad \alpha _\varepsilon :=\alpha + \varepsilon ,\quad \beta _\varepsilon :=\beta + \varepsilon , \end{aligned}$$

thereby ensuring that the modified parameters continue to satisfy the condition in (2.13) and (2.15). Noting that the Caffarelli–Kohn–Nirenberg inequalities are invariant under scaling, we may assume without loss of generality that $\Omega$ is contained in the unit ball (so that $|x|<1$). Then, by letting $\varepsilon \rightarrow 0$ and applying the monotone convergence theorem, the desired inequality follows. Thus, the corollary is established. $\square$

### Proof of Theorem 3

By Theorem 1, we know that if $\Omega \subseteq {\mathbb {R}}^{N}$ is an arbitrary open set, then for every $u \in C^{\infty }_c ( \Omega )$, there holds

3.15 $$\begin{aligned} \int _{\Omega } \omega ^{r} (x) | u (x) |^{r} \textrm{d}x&\leqslant \frac{r}{\kappa _+} \int _{\Omega } | x | \omega ^{r} (x) | u |^{r- 1} \left| \nabla u (x) \right| \textrm{d}x, \end{aligned}$$

provided that $N+ r\gamma > 0$ if $\gamma < 0$ and with $\kappa _+$ given by $\kappa _+ = \left( N+ \gamma r\right)$ if $\gamma < 0$ and $\kappa _+ = N$ if $\gamma > 0$. We introduce real parameters $\alpha , \beta \in {\mathbb {R}}$ and rewrite (3.7) under the form

$$\begin{aligned} \int _{\Omega } \omega ^{r} | u |^{r} \textrm{d}x&\leqslant \frac{r}{\kappa _+} \int _{\Omega } \left( | x | \omega ^{r} \right) ^{\alpha } \cdot \left| \nabla u \right| \cdot \left( \left( | x | \omega ^{r} \right) ^{\beta } | u | \right) ^{r- 1} \textrm{d}x, \end{aligned}$$

which is equivalent to (3.15) as soon as we chose $\alpha , \beta \in {\mathbb {R}}$ such that $\alpha + \beta \left( r- 1 \right) = 1$.

Now, if $p, q\in [1, + \infty ]$ are such that $1 / p+ \left( r- 1 \right) / q= 1$, then by classical (two-factors) Hölder inequality, we infer that

3.16 $$\begin{aligned} \left\| \omega | u | \right\| _{L^{r} ( \Omega )}^{r}&\leqslant \frac{r}{\kappa _+} \left\| \, \left( | x | \omega ^{r} \right) ^{\alpha } \cdot \left| \nabla u \right| \, \right\| _{L^{p} ( \Omega )} \; \cdot \; \left\| \left( \left( | x | \omega ^{r} \right) ^{\beta } | u | \right) ^{r- 1} \right\| _{L^{\frac{q}{\left. \left( r- 1 \right. \right) }} ( \Omega )} \nonumber \\&=\frac{r}{\kappa _+} \left\| \, | x |^{\alpha } \omega ^{\alpha r} \left| \nabla u \right| \, \right\| _{L^{p} ( \Omega )} \; \cdot \; \left\| \, | x |^{\beta } \omega ^{\beta r} | u | \, \right\| _{L^{q} ( \Omega )}^{\left( r- 1 \right) }, \end{aligned}$$

provided that the constraints (2.19) are met. However, the previous relation (3.16) is nothing but (2.18); therefore, we are done. $\square$

### Proof of Corollary 3

Let us start with the first-order interpolation inequality (2.18). If $\alpha + \beta \left( r- 1 \right) = 1$ and $\frac{1}{p} + \frac{1}{q} =1$ then

$$\begin{aligned} \left\| \omega | u | \right\| _{L^{r} ( \Omega )} \leqslant \left( \frac{r}{\kappa _+} \right) ^{1 / r} \left\| \, | x |^{\alpha } \omega ^{\alpha r} \left| \nabla u \right| \, \right\| _{L^{p}( \Omega )}^{1 / r} \; \cdot \; \left\| \, | x |^{\beta } \omega ^{\beta r} | u | \, \right\| _{L^{q} ( \Omega )}^{1 - 1 / r} \quad \forall u \in C^{\infty }_c ( \Omega ). \end{aligned}$$

But $\omega (x) = (\lambda + | x |^{\mu })^{\gamma / \mu } \rightarrow | x |^{\gamma }$ pointwise in ${\mathbb {R}}^{N}$ when $\lambda \rightarrow 0$, therefore by monotone convergence theorem if $\gamma < 0$ and dominated convergence theorem if $\gamma > 0$, we get that

$$\begin{aligned} \Vert | x |^{\gamma } | u | \Vert _{L^{r} ( \Omega )}\leqslant &   \left( \frac{r}{\kappa _+} \right) ^{1 / r} \left\| \, | x |^{\alpha \left( 1 + \gamma r\right) } \left| \nabla u \right| \, \right\| _{L^{p} ( \Omega )}^{1 / r} \; \\    &   \cdot \; \left\| \, | x |^{\beta \left( 1 + \gamma r\right) } | u | \, \right\| _{L^{q} ( \Omega )}^{1 - 1 / r} \quad \forall u \in C^{\infty }_c ( \Omega ). \end{aligned}$$

We first assume $\gamma \ne -1/r$. Then, with ${\tilde{\alpha }} :=\alpha \left( 1 + \gamma r\right)$ and ${\tilde{\beta }} :=\beta \left( 1 + \gamma r\right)$ we get

$$\begin{aligned} \Vert | x |^{\gamma } | u | \Vert _{L^{r} ( \Omega )} \leqslant \left( \frac{r}{\kappa _+} \right) ^{1 / r} \left\| \, | x |^{{\tilde{\alpha }}} \left| \nabla u \right| \, \right\| _{L^{p} ( \Omega )}^a \; \cdot \; \left\| \, | x |^{{\tilde{\beta }}} | u | \, \right\| _{L^{q} ( \Omega )}^{1 - a} \end{aligned}$$

with ${\tilde{\alpha }}$ and ${\tilde{\beta }}$ such that ${\tilde{\alpha }} + {\tilde{\beta }} \left( r- 1 \right) =1 + \gamma r$. Renaming back ${\tilde{\alpha }}$ and ${\tilde{\beta }}$ with $\alpha$ and $\beta$ we conclude. Eventually, if $\gamma = -1/r$ we argue as in the proof of Corollary 2. $\square$

### Proof of Theorem 4

By the radial integration by parts formula with boundary reminder (Theorem 1.*i*), we know that if $\Omega \subseteq {\mathbb {R}}^{N}$ is a bounded open set with a Lipschitz boundary, then for every $u \in C^{\infty } ( {\overline{\Omega }})$, there holds

3.17 $$\begin{aligned} \kappa _+ \int _{\Omega } \omega ^{r} | u |^{r} \textrm{d}x&\leqslant r\int _{\Omega } | x | \omega ^{r} | u |^{r- 1} \left| \nabla u \right| \textrm{d}x + \int _{\partial \Omega } \omega ^{r} | u |^{r} (\xi \cdot \varvec{\nu }) \textrm{d}\xi \end{aligned}$$

provided that $N+ r\gamma > 0$ if $\gamma < 0$ and with $\kappa _+ > 0$ given by $\kappa _+ = \left( N+ \gamma r\right)$ if $\gamma < 0$ and $\kappa _+ = N$ if $\gamma > 0$. We introduce real parameters $a, \alpha , \beta \in {\mathbb {R}}$ and rewrite (3.17) under the form

$$\begin{aligned} \int _{\Omega } \omega ^{r} | u |^{r} \textrm{d}x&\leqslant \frac{r}{\kappa _+} \int _{\Omega } \left( | x | \omega ^{r} \right) ^{\alpha } \cdot \left| \nabla u \right| \cdot \left( \left( | x | \omega ^{r} \right) ^{\beta } | u | \right) ^{r- 1} \textrm{d}x \\&\quad + \frac{1}{\kappa _+} \int _{\partial \Omega } \omega ^{r} | u |^{r} (\xi \cdot \varvec{\nu }) \textrm{d}\xi \end{aligned}$$

which is equivalent to (3.17) as soon as we chose $\alpha , \beta \in {\mathbb {R}}$ such that $\alpha + \beta \left( r- 1 \right) = 1$.

Now, if $p, q\in [1, + \infty ]$ are such that $1 / p+ 1 / q= 1$, then by classical (two-factors) Hölder inequality, we infer that

3.18 $$\begin{aligned} \left\| \omega | u | \right\| _{L^{r} ( \Omega )}^{r}&\leqslant \frac{r}{\kappa _+} \left\| \, \left( | x | \omega ^{r} \right) ^{\alpha } \cdot \left| \nabla u \right| \, \right\| _{L^{p} ( \Omega )} \; \cdot \; \left\| \left( \left( | x | \omega ^{r} \right) ^{\beta } | u | \right) ^{r- 1} \right\| _{L^{\frac{q}{\left. \left( r- 1 \right. \right) }} ( \Omega )} \nonumber \\&\quad + \frac{1}{\kappa _+} \int _{\partial \Omega } \omega ^{r} | u |^{r} (\xi \cdot \varvec{\nu }) \textrm{d}\xi \nonumber \\&=\frac{r}{\kappa _+} \left\| \, | x |^{\alpha } \omega ^{\alpha r} \left| \nabla u \right| \, \right\| _{L^{p} ( \Omega )} \; \cdot \; \left\| \, | x |^{\beta } \omega ^{\beta r} | u | \, \right\| _{L^{q} ( \Omega )}^{\left( r- 1 \right) } \nonumber \\&\quad + \frac{1}{\kappa _+} \int _{\partial \Omega } \omega ^{r} | u |^{r} (\xi \cdot \varvec{\nu }) \textrm{d}\xi , \end{aligned}$$

provided that the constraints in (2.25) are met. By the classical inequality $| a + b |^{1 / r} \leqslant 2^{1 / r} \left( | a |^{1 / r} + | b |^{1 / r} \right)$, which is valid for every $r> 0$, we get (2.24). $\square$

### Proof of Corollary 4

Let us start with the first-order interpolation inequality (2.24). As before, since $\omega (x) = (\lambda + | x |^{\mu })^{\gamma / \mu } \rightarrow | x |^{\gamma }$ pointwise in ${\mathbb {R}}^{N}$ when $\lambda \rightarrow 0$, by monotone convergence theorem if $\gamma < 0$ and dominated convergence theorem if $\gamma > 0$, we get that

3.19 $$\begin{aligned} \Vert | x |^{\gamma } | u | \Vert _{L^{r} ( \Omega )}&\leqslant \left( \frac{2 r}{\kappa _+} \right) ^{1 / r} \left\| \, | x |^{\alpha \left( 1 + \gamma r\right) } \left| \nabla u \right| \, \right\| _{L^{p} ( \Omega )}^{1 / r} \; \cdot \; \left\| \, | x |^{\beta \left( 1 + \gamma r\right) } \omega ^{\beta r} | u | \, \right\| _{L^{q} \left( \Omega \right) }^{1 - 1 / r} \nonumber \\&\quad + \left( \frac{2}{\kappa _+} \right) ^{1 / r} \left( \int _{\partial \Omega } | x |^{\gamma r} | u |^{r} (\xi \cdot \varvec{\nu }) \textrm{d}\xi \right) ^{1 / r} . \end{aligned}$$

We first assume $\gamma \ne -1/r$. Then, with ${\tilde{\alpha }} :=\alpha \left( 1 + \gamma r\right)$ and ${\tilde{\beta }} :=\beta \left( 1 + \gamma r\right)$ we get

3.20 $$\begin{aligned} \Vert | x |^{\gamma } | u | \Vert _{L^{r} ( \Omega )}&\leqslant \left( \frac{r}{\kappa _+} \right) ^{1 / r} \left\| \, | x |^{{\tilde{\alpha }}} \left| \nabla u \right| \, \right\| _{L^{p} ( \Omega )}^a \; \cdot \; \left\| \, | x |^{{\tilde{\beta }}} | u | \, \right\| _{L^{q} \left( \Omega \right) }^{1 - a} \nonumber \\&\quad + \left( \frac{2}{\kappa _+} \right) ^{1 / r} \left( \int _{\partial \Omega } | x |^{\gamma r} | u |^{r} (\xi \cdot \varvec{\nu }) \textrm{d}\xi \right) ^{1 / r}, \end{aligned}$$

with ${\tilde{\alpha }}$ and ${\tilde{\beta }}$ such that ${\tilde{\alpha }} + {\tilde{\beta }} \left( r- 1 \right) =1 + \gamma r$. Renaming back ${\tilde{\alpha }}$ and ${\tilde{\beta }}$ with $\alpha$ and $\beta$, we conclude. Eventually, if $\gamma = -1/r$ we argue as in the proof of Corollary 2. $\square$

## Data Availability

No datasets were generated or analysed during the current study.
